# Supplementary material for: Influenza A viruses are transmitted via the air from the nasal respiratory epithelium of ferrets
Source: Nat Commun. 2020 Feb 7;11:766. doi: 10.1038/s41467-020-14626-0 (PMC7005743; doi:10.1038/s41467-020-14626-0)
Supplement: Supplementary file 1 — Supplementary Information [file 41467_2020_14626_MOESM1_ESM.pdf]

Supplementary information for

Influenza A viruses are transmitted via the air from the nasal respiratory epithelium of ferrets

Mathilde Richard [a], Judith M.A. van den Brand [a], Theo M. Bestebroer [a], Pascal Lexmond [a], Dennis de Meulder [a], Ron A.M. Fouchier [a], Anice C. Lowen [b,c] and Sander Herfst [a,d].

[a] Department of Viroscience, Erasmus MC University Medical Center, Rotterdam, the Netherlands. Center for Research on Influenza Pathogenesis (CRIP) Center of Excellence for Influenza Research and Surveillance (CEIRS).

[b] Department of Microbiology and Immunology, Emory University School of Medicine, Atlanta, GA 30322, USA

[c] Emory-UGA Center of Excellence for Influenza Research and Surveillance (CEIRS), Atlanta, GA 30322, USA

[d] Corresponding author: s.herfst@erasmusmc.nl

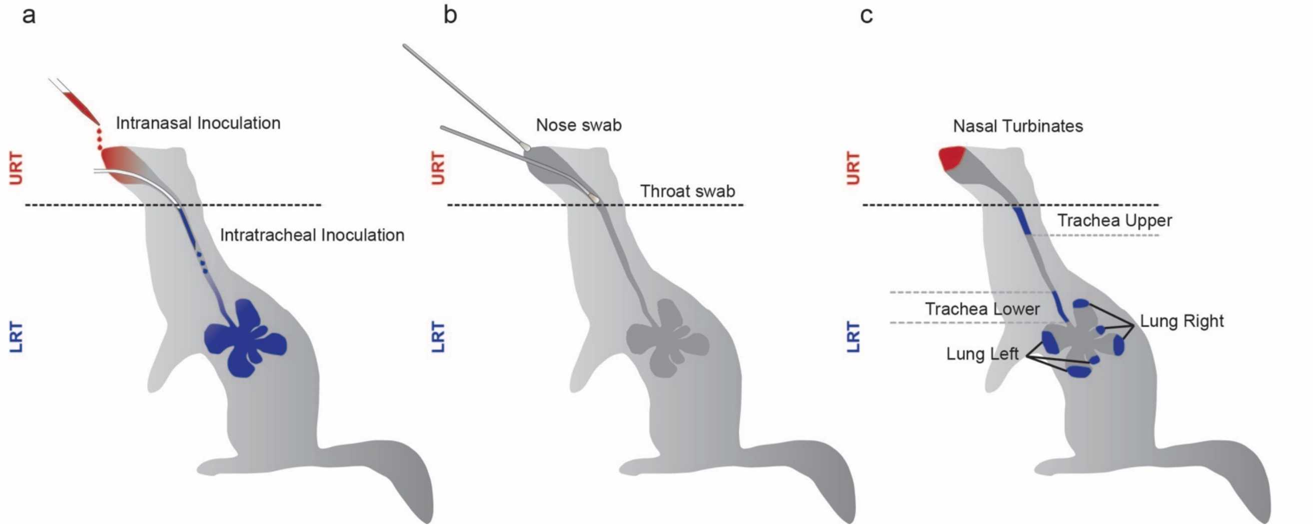

Supplementary figure 1. Anatomic sites of inoculation and sampling within the ferret respiratory tract. Anatomic sites accessed upon A. Inoculation, B. Swab collection and C. Tissue collection are shown schematically. The upper respiratory tract (URT) is composed of the nasal turbinates, paranasal sinuses, pharynx and larynx. The lower respiratory tract (LRT) is composed of the trachea, bronchus and lung (bronchioles and alveoli). The distinction between upper and lower respiratory tract is not based on a strict anatomical designation but on differences in physiology and mucociliary transport going downwards from the nasal turbinates and upwards from the terminal bronchioles (through bronchus/trachea) to the pharynx for clearance by entrance into the gastrointestinal tract [1].

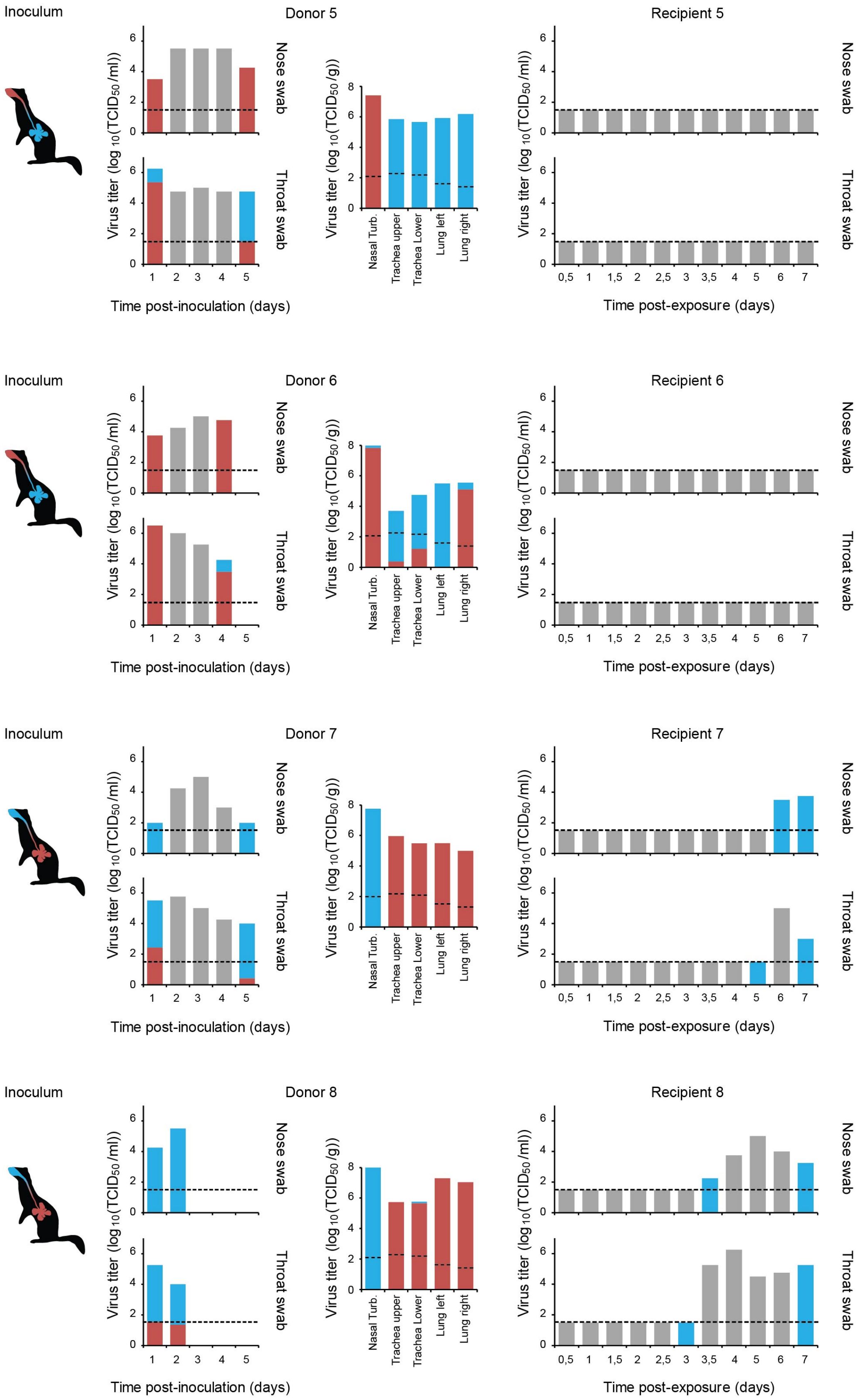
Inoculum
Donor 5
Recipient 5
Virus titer (log10(TCID50 /ml))
Nose swab
Throat swab
Virus titer (log10(TCID50 /g))
Nasal Turb.
Trachea upper
Trachea Lower
Lung left
Lung right
0
2
4
6
8
1
2
3
4
5
0,5
1
1,5
2
2,5
3
3,5
4
5
6
7
Time post-inoculation (days)
Time post-exposure (days)
Inoculum
Donor 6
Recipient 6
Virus titer (log10(TCID50 /ml))
Nose swab
Throat swab
Virus titer (log10(TCID50 /g))
Nasal Turb.
Trachea upper
Trachea Lower
Lung left
Lung right
Time post-inoculation (days)
Time post-exposure (days)
Inoculum
Donor 7
Recipient 7
Virus titer (log10(TCID50 /ml))
Nose swab
Throat swab
Virus titer (log10(TCID50 /g))
Nasal Turb.
Trachea upper
Trachea Lower
Lung left
Lung right
Time post-inoculation (days)
Time post-exposure (days)
Inoculum
Donor 8
Recipient 8
Virus titer (log10(TCID50 /ml))
Nose swab
Throat swab
Virus titer (log10(TCID50 /g))
Nasal Turb.
Trachea upper
Trachea Lower
Lung left
Lung right
Time post-inoculation (days)
Time post-exposure (days)

Supplementary figure 2. The A/H1N1 virus was transmitted from the upper respiratory tract of ferrets.

Donor ferrets 5 and 6 were inoculated intranasally with 10^5 $TCID_{50}$ of the A/H1N1 virus (shown in red) and intratracheally with 10^5 $TCID_{50}$ of the A/H1N1$_{var}$ virus (shown in blue). Donor ferrets 7 and 8 were inoculated with the opposite placement of viruses. Recipient ferrets were added to the opposite cage at 4 hpi. At the day that transmission to recipient ferrets was observed, or the latest at 5dpi, donor ferrets were euthanized and tissues from the respiratory tract (nasal turbinates (Nasal turb.), the upper part of the trachea, the lower part of the trachea, the left and the right lungs) were harvested. Virus titers in the swabs of donor and recipient ferrets and in the tissues of donor ferrets were determined by $TCID_{50}$ assay and are indicated on the y-axis. The limit of detection of the virus titrations is shown by the dotted line. For donor ferrets, swabs collected at 1 dpi, on the day of transmission or the latest at 5 dpi, and from tissues of the respiratory tract were processed for next-generation sequencing. For recipient ferrets, the first and the last samples that were positive (threshold value in RT-qPCR (Ct value) <35) were processed for next-generation sequencing. The proportions of untagged (red) and tagged (blue) viruses, as determined by next-generation sequencing, are indicated by the coloured bars. The grey bars correspond to samples that were not included in the next-generation sequencing. Source data are provided as a Source Data file.

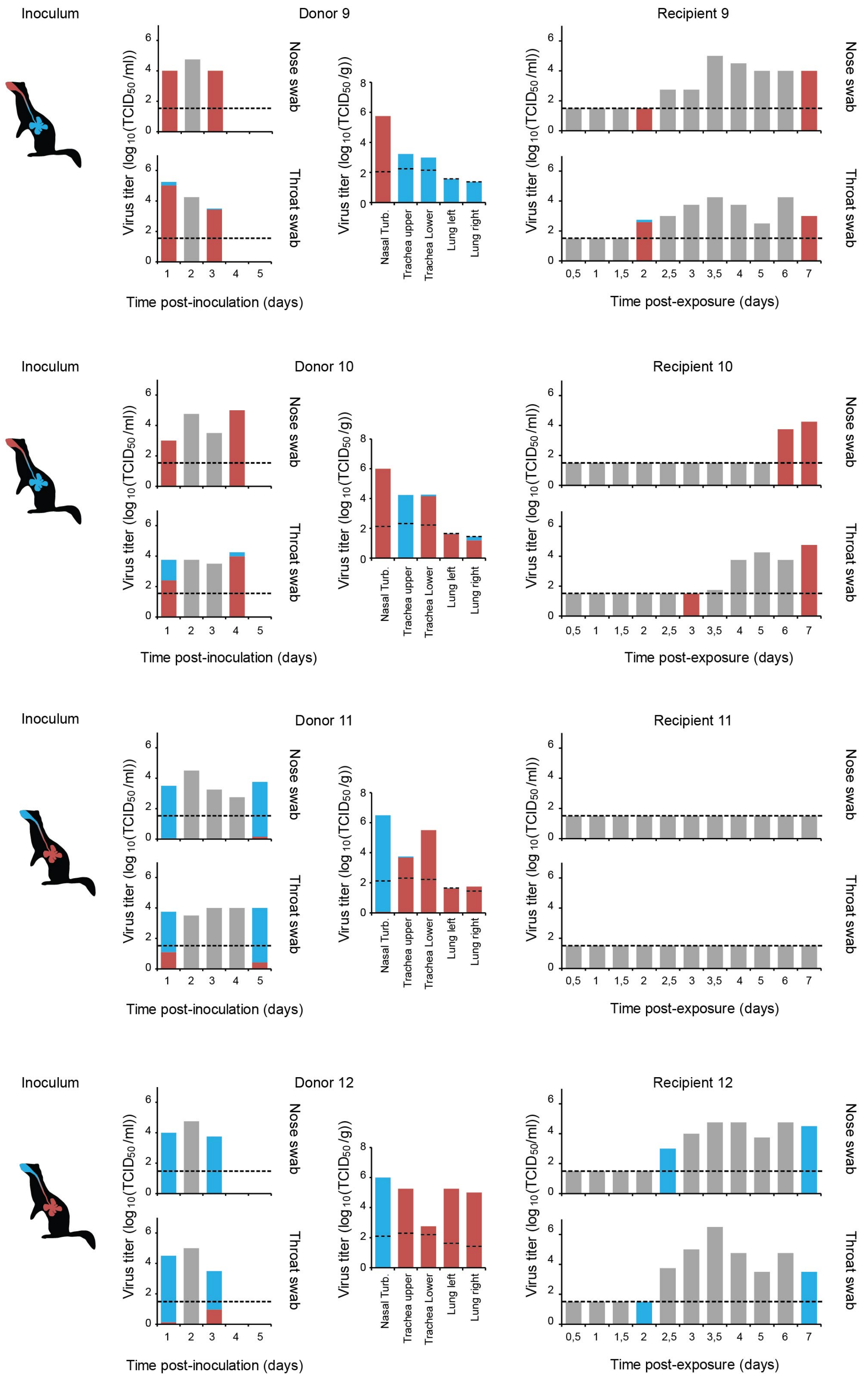
Inoculum
Donor 9
Recipient 9
Inoculum
Donor 10
Recipient 10
Inoculum
Donor 11
Recipient 11
Inoculum
Donor 12
Recipient 12
Virus titer (log10(TCID50/ml))
Virus titer (log10(TCID50/g))
Nose swab
Throat swab
Nasal Turb.
Trachea upper
Trachea Lower
Lung left
Lung right
Time post-inoculation (days)
Time post-exposure (days)
0,5
1
1,5
2
2,5
3
3,5
4
5
6
7

Supplementary figure 3. The A/H3N2 virus was transmitted from the upper respiratory tract of ferrets.

Donor ferrets 9 and 10 were inoculated intranasally with 10^5 $TCID_{50}$ of the A/H3N2 virus (shown in red) and intratracheally with 10^5 $TCID_{50}$ of the A/H3N2$_{var}$ virus (shown in blue). Donor ferrets 11 and 12 were inoculated with the opposite placement of viruses. Recipient ferrets were added to the opposite cage at 4 hpi. At the day that transmission to recipient ferrets was observed, or the latest at 5dpi, donor ferrets were euthanized and tissues from the respiratory tract (nasal turbinates (Nasal turb.), the upper part of the trachea, the lower part of the trachea, the left and the right lungs) were harvested. Virus titers in the swabs of donor and recipient ferrets and in the tissues of donor ferrets were determined by $TCID_{50}$ assay and are indicated on the y-axis. The limit of detection of the virus titrations is shown by the dotted line. For donor ferrets, swabs collected at 1 dpi, on the day of transmission or the latest at 5 dpi, and from tissues of the respiratory tract were processed for next-generation sequencing. For recipient ferrets, the first and the last samples that were positive (threshold value in RT-qPCR (Ct value) <35) were processed for next-generation sequencing. The proportions of untagged (red) and tagged (blue) viruses, as determined by next-generation sequencing, are indicated by the coloured bars. The grey bars correspond to samples that were not included in the next-generation sequencing. Source data are provided as a Source Data file.

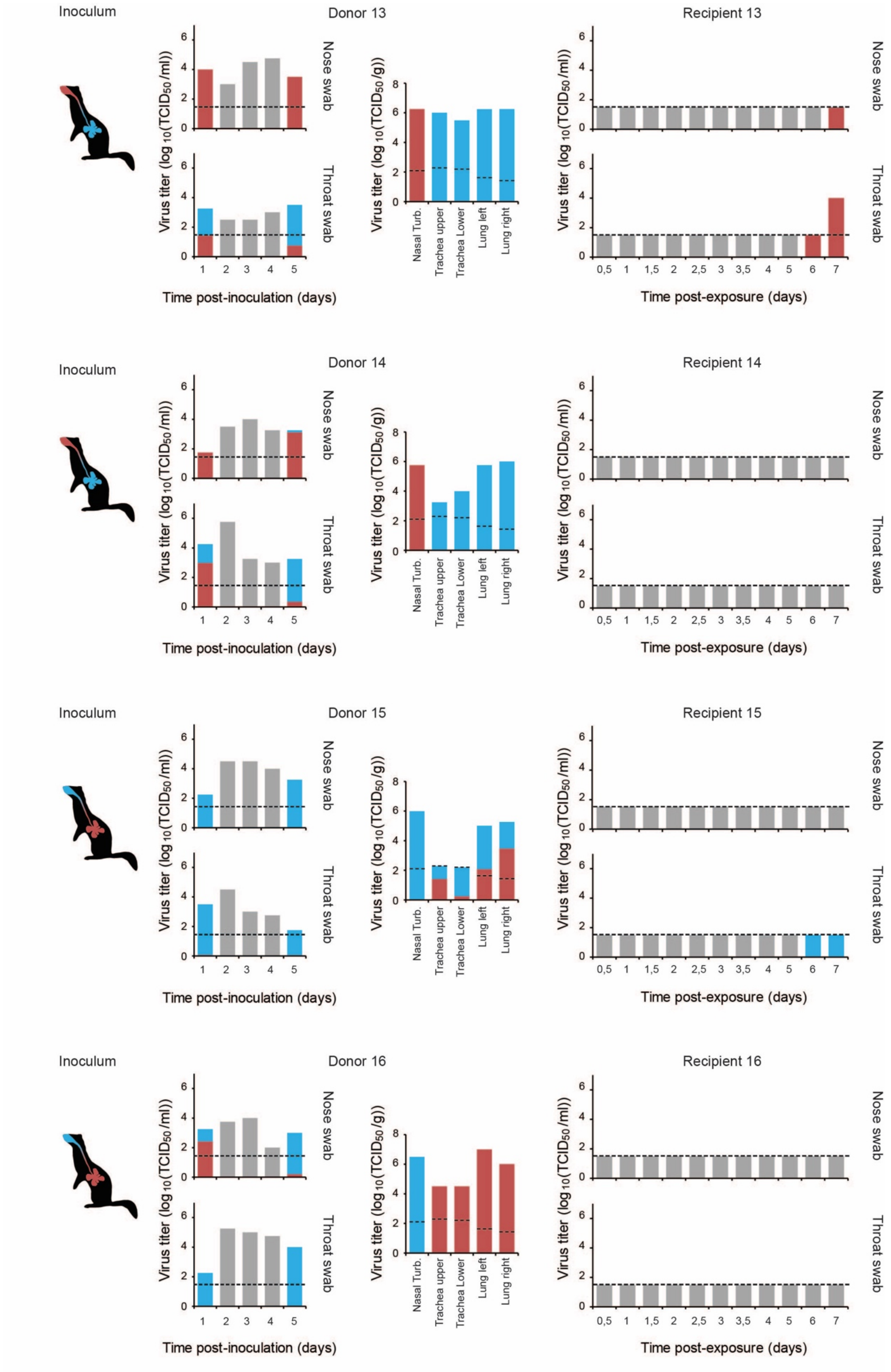
Inoculum
Donor 13
Recipient 13
Inoculum
Donor 14
Recipient 14
Inoculum
Donor 15
Recipient 15
Inoculum
Donor 16
Recipient 16
Virus titer (log 10(TCID50 /ml))
Virus titer (log 10(TCID50 /g))
Nose swab
Throat swab
Nasal Turb.
Trachea upper
Trachea Lower
Lung left
Lung right
Time post-inoculation (days)
Time post-exposure (days)

Supplementary figure 4. The A/H5N1$_{AT}$ virus was transmitted from the upper respiratory tract of ferrets.

Donor ferrets 13 and 14 were inoculated intranasally with 10^5 $TCID_{50}$ of the A/H5N1$_{AT}$ virus (shown in red) and intratracheally with 10^5 $TCID_{50}$ of the A/ H5N1$_{AT\text{-}var}$ virus (shown in blue). Donor ferrets 15 and 16 were inoculated with the opposite placement of viruses. Recipient ferrets were added to the opposite cage at 4 hpi. At the day that transmission to recipient ferrets was observed, or the latest at 5dpi, donor ferrets were euthanized and tissues from the respiratory tract (nasal turbinates (Nasal turb.), the upper part of the trachea, the lower part of the trachea, the left and the right lungs) were harvested. Virus titres in the swabs of donor and recipient ferrets and in the tissues of donor ferrets were determined by $TCID_{50}$ assay and are indicated on the y-axis. The limit of detection of the virus titrations is shown by the dotted line. For donor ferrets, swabs collected at 1 dpi, on the day of transmission or the latest at 5 dpi, and from tissues of the respiratory tract were processed for next-generation sequencing. For recipient ferrets, the first and the last samples that were positive (threshold value in RT-qPCR (Ct value) <35) were processed for next-generation sequencing. The proportions of untagged (red) and tagged (blue) viruses, as determined by next-generation sequencing, are indicated by the coloured bars. The grey bars correspond to samples that were not included in the next-generation sequencing. Source data are provided as a Source Data file.

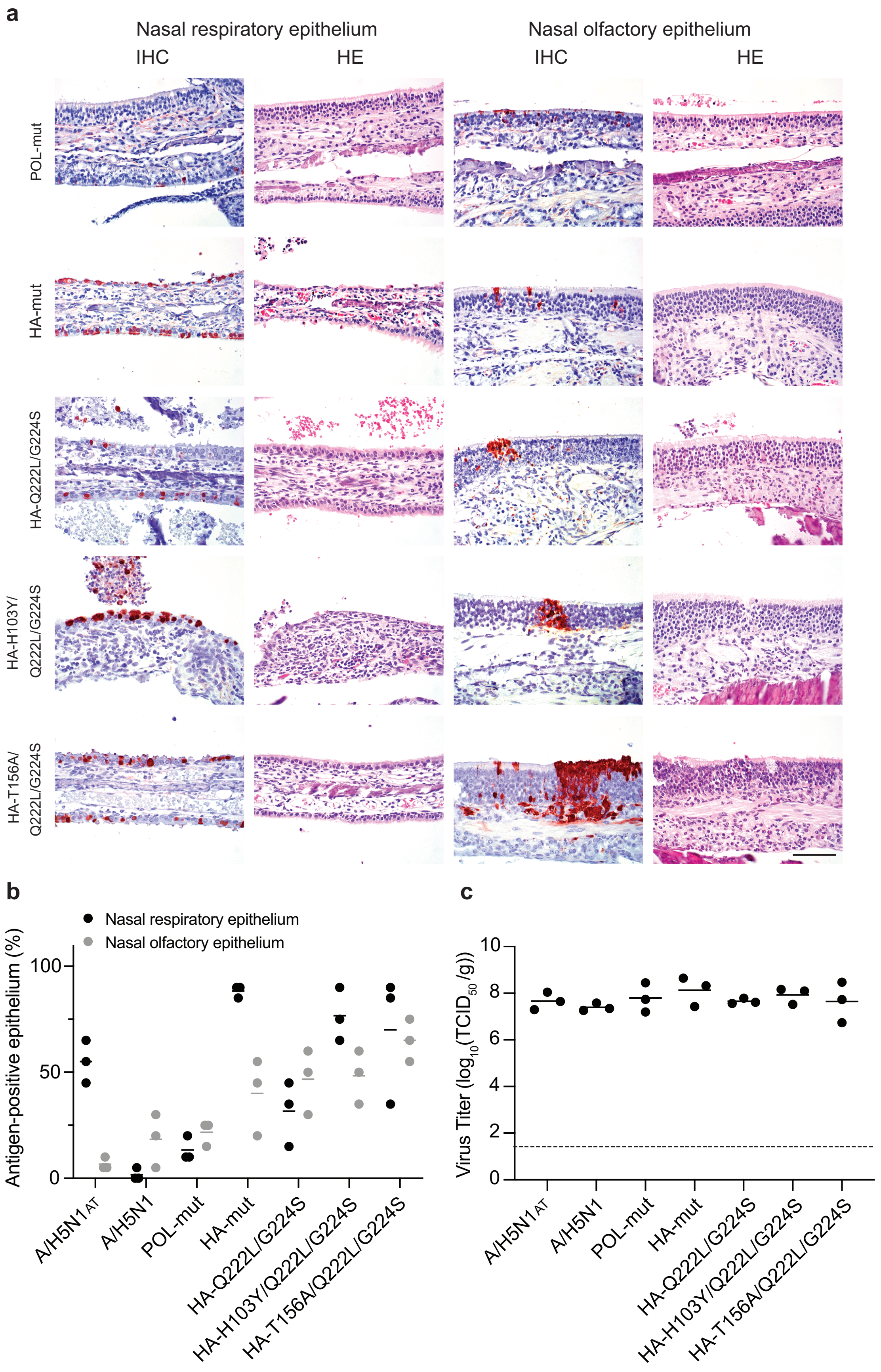

a
Nasal respiratory epithelium
Nasal olfactory epithelium
IHC
HE
IHC
HE
POL-mut
HA-mut
HA-Q222L/G224S
HA-H103Y/Q222L/G224S
HA-T156A/Q222L/G224S
b
Nasal respiratory epithelium
Nasal olfactory epithelium
Antigen-positive epithelium (%)
100
50
0
A/H5N1AT
A/H5N1
POL-mut
HA-mut
HA-Q222L/G224S
HA-H103Y/Q222L/G224S
HA-T156A/Q222L/G224S
c
Virus Titer (log10(TCID50/g))
10
8
6
4
2
0
A/H5N1AT
A/H5N1
POL-mut
HA-mut
HA-Q222L/G224S
HA-H103Y/Q222L/G224S
HA-T156A/Q222L/G224S

Supplementary figure 5. The tropism for ferret nasal respiratory epithelium is determined by adaptive substitutions in the HA gene.

Representative pictures of ferret nasal respiratory and nasal olfactory epithelia 2 days after intranasal inoculation with either an A/H5N1 virus with polymerase substitutions (POL-mut containing substitutions PB2-E627K, PB1-H99Y, PB1-I368V, NP-R99K, NP-S345N), an A/H5N1 virus with HA substitutions (HA-mut, containing HA-H103Y, HA-T156A, HA-Q222L, HA-G224S), A/H5N1 containing HA-Q222L and HA-G224S (Q222L/G224S), an A/H5N1 virus containing HA-H103Y, HA-Q222L and HA-G224S (H103Y/Q222L/G224S) or an A/H5N1 virus containing HA-T156A, HA-Q222L and HA-G224S (T156A/Q222L/G224S). Influenza A virus nucleoprotein expression was determined by immunohistochemistry (IHC) and is shown as a red stain. HE: hematoxylin-eosin stain. Scale bar 50 µm. **b.** Percentages epithelium that was nucleoprotein antigen-positive, as determined by IHC, were blindly assessed in the nasal respiratory epithelium (black) and nasal olfactory epithelium (light grey) of three ferrets inoculated with the respective viruses. Individual percentages are shown. Means are depicted by the horizontal lines. c. Individual virus titers in the homogenized nasal turbinates (containing both nasal respiratory and olfactory epithelia) was determined by end point titration in MDCK. Means are depicted by the horizontal lines. The limit of detection of the virus titration is shown by the dotted line. Source data are provided as a Source Data file.

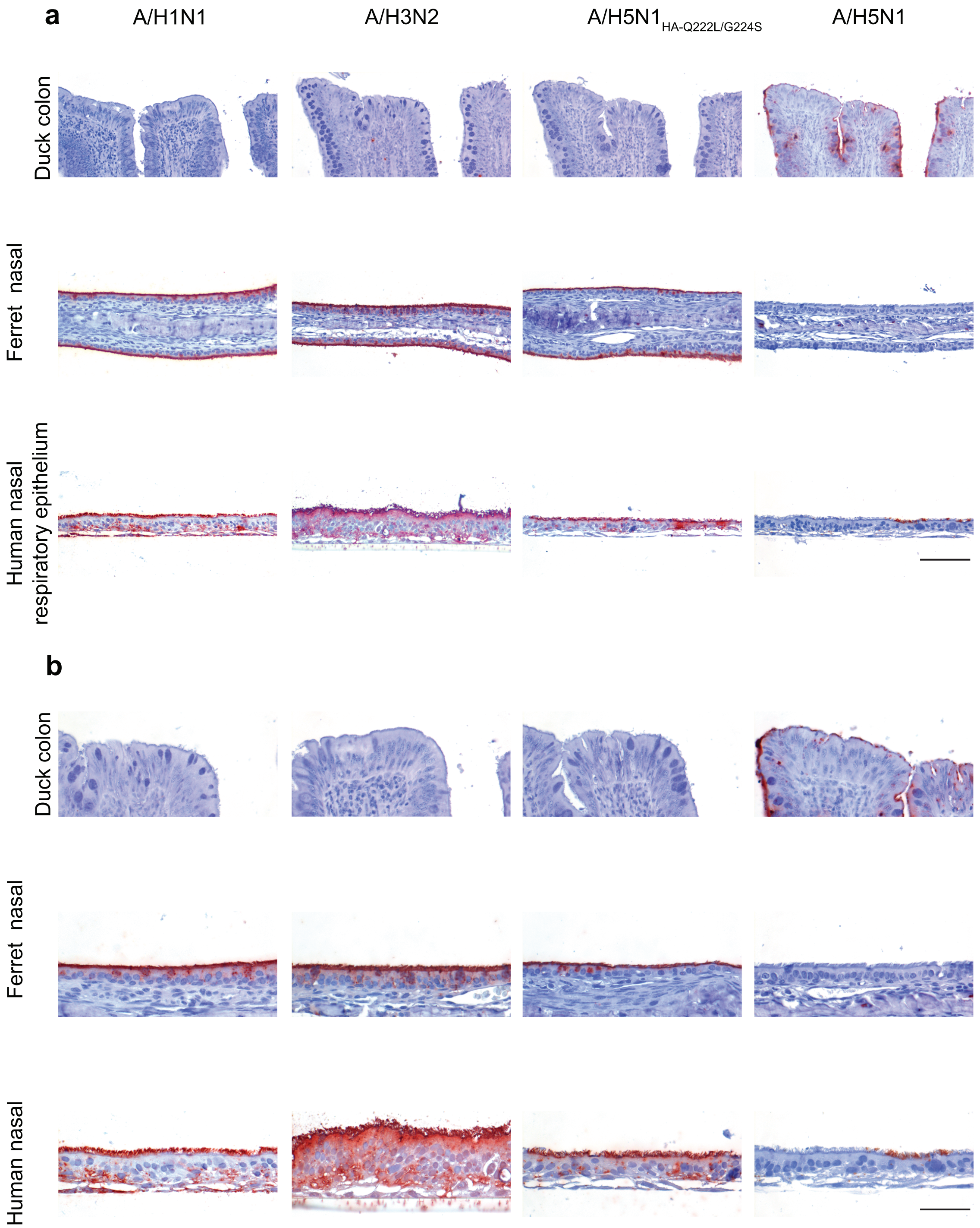
a
A/H1N1
A/H3N2
A/H5N1HA-Q222L/G224S
A/H5N1
Duck colon
Ferret nasal
Human nasal respiratory epithelium
b
Duck colon
Ferret nasal
Human nasal

Supplementary figure 6. Airborne transmissible influenza A viruses bind abundantly to ferret and human nasal respiratory epithelium.

Virus binding of A/H1N1, A/H3N2, A/H5N1$_{AT}$ or A/H5N1 viruses on duck colon, ferret nasal respiratory epithelium, and primary culture of human nasal respiratory epithelium (Mucilair^{TM}) was assessed by virus histochemistry. Ferret nasal respiratory epithelium and duck colon tissues were used as controls for the binding of human and avian influenza viruses respectively Red staining depicts the binding of FITC-labelled influenza viruses to the epithelium of the different tissues. **a.** Scale bar 100 µm. **b.** Scale bar 50 µm.

Supplementary Table 1. Inoculation scheme of donor ferrets for transmission experiments.

Number of donor ferret	Virus inoculated intranasally	Virus inoculated intratracheally
Donor 1-2-5-6	A/H1N1	A/H1N1$_{var}$
Donor 3-4-7-8	A/H1N1$_{var}$	A/H1N1
Donor 9-10	A/H3N2	A/H3N2$_{var}$
Donor 11-12	A/H3N2$_{var}$	A/H3N2
Donor 13-14	A/H5N1$_{AT}$	A/H5N1$_{AT\text{-}var}$
Donor 15-16	A/H5N1$_{AT\text{-}var}$	A/H5N1$_{AT}$

Supplementary Table 2. Primers used in the study.

**Primers to make A/$H1N1_{var}$**	
PB2 C273T F	CTCTGGAGCAAAACAAATGATGCTGGATCAGAC
PB2 C273T R	GTCTGATCCAGCATCATTTGTTTTGCTCCAGAG
PB1 T288C F	GCACAAACAGACTGTGTCCTAGAGGCTATGGCTTTC
PB1 T288C R	GAAAGCCATAGCCTCTAGGACACAGTCTGTTTGTGC
PA C360T F	CTGATTTGTATGATTATAAAGAGAACCGGTTC
PA C360T R	GAACCGGTTCTCTTTATAATCATACAAATCAG
HA C305T F	CTCTCCACAGCAAGTTCATGGTCCTACATTG
HA C305T R	CAATGTAGGACCATGAACTTGCTGTGGAGAG
NP A351G F	GAAGAGTAGACGGGAAGTGGATGAGAGAACTC
NP A351G R	GAGTTCTCTCATCCACTTCCCGTCTACTCTTC
NA G336A F	CAGTAAAGACAACAGTATAAGAGTCGGTTCC
NA G336A R	GGAACCGACTCTTATACTGTTGTCTTTACTG
M G295A F	GGGAATGGGGACCCAAACAACATGGATAGAG
M G295A R	CTCTATCCATGTTGTTTGGGTCCCCATTCCC
NS C341T F	GAGACTGGTTCATGCTTATGCCTAGGCAAAAG
NS C341T R	CTTTTGCCTAGGCATAAGCATGAACCAGTCTC
**Primers to make A/$H3N2_{var}$**	
PB2 C354T, C360T F	GTGACAAGTACGGTTCACTATCCAAAAGTATACAAG
PB2 C354T, C360T R	GTGACAAGTACGGTTCACTATCCAAAAGTATACAAG
PB1 A540G F	CTCAAGGATGTGATGGAGTCAATGGATAAAGAGG
PB1 A540G R	CCTCTTTATCCATTGACTCCATCACATCCTTGAG
PA G333A, A342G F	GGAGCTGAGAAACCAAAGTTTCTGCCAGATTTGTATG
PA G333A, A342G R	CATACAAATCTGGCAGAAACTTTGGTTTCTCAGCTCC
HA T308C, C311A, C314T F	GGAGACCCTCATTGTGACGGATTTCAAAATAAGGAATGGGAC
HA T308C, C311A, C314T R	GTCCCATTCCTTATTTTGAAATCCGTCACAATGAGGGTCTCC
HA A464T, C467G, T470A R	GAGCTTGTTCCATTCTGTGCCACACCAGTCCAATTGAAGC
HA A464T, C467G, T470A F	GCTTCAATTGGACTGGTGTGGCACAGAATGGAACAAGCTC
NP C537T, T538A, C539G F	GAATGGATCCCAGAATGTGTAGTCTGATGCAGGGCTC
NP C537T, T538A, C539G R	GAGCCCTGCATCAGACTACACATTCTGGGATCCATTC
NA C418G, T421A, A424C F	CAAGTGTTATCAATTTGCGCTAGGCCAGGGAACAACACTAAAC
NA C418G, T421A, A424C R	GTTTAGTGTTGTTCCCTGGCCTAGCGCAAATTGATAACACTTG
M G586A F	GCCTCATATACAATAGAATGGGGGCTGTAAC
M G586A R	GTTACAGCCCCCATTCTATTGTATATGAGGC
NS C329T, C335T, A341G F	GGAATTGTCAAGAAATTGGTTTATGCTGATGCCCAAGCAAA
NS C329T, C335T, A341G R	TTTGCTTGGGCATCAGCATAAACCAATTTCTTGACAATTCC
**Primers to make A/$H5N1_{var}$**	
PB2 A339G F	GGACTGCACTTGTCGTCGGCCCATTCCTATTCC
PB2 A339G R	GGAATAGGAATGGGCCGACGACAAGTGCAGTCC

**Primers to make A/H5N1$_{AT}$**	
PB2 E627K F	GCAGCAGCCCCACCAAAGCAGAGCAGAA
PB2 E627K R	TTCTGCTCTGCTTTGGTGGGGCTGCTGC
PB1 H99Y F	GCTTTCTTGAACAATCCTACCCAGGGATTTGAAA
PB1 H99Y R	TTTCAAATCCCTGGGTAGGATTGTTCAAGAAAGC
NP R99K F	AGGTCCAATTTATCGGAAGAGAGACGGAAAATGGG
NP R99K R	CCCATTTTCCGTCTCTCTTCCGATAAATTGGACCT
NP S345N F	GAGGACCTTAGAGTTTCAAATTCATCAGAGGGACAAGA
NP S345N R	TCTTGTCCCTCTGATGAATTTGAAACTCTAAGGTCCTC
HA H103Y F	GGAGTTTCAACGACTATGAAGAACTGAAATACCTATTGAGCAGAA
HA H103Y R	TTCTGCTCAATAGGTATTTCAGTTCTTCATAGTCGTTGAAACTCC
HA T156A F	TATGGCTTATCAAAAAGAACAGTGCATACCCAACAATAAAGAAAAGC
HA T156A R	GCTTTTCTTTATTGTTGGGTATGCACTGTTCTTTTTGATAAGCCATA
HA Q222L, G224S F	TAGATCCAAAGTAAACGGGCTAAGTAGCAGGATGGAGTTCTTCTGGAC
HA Q222L, G224S R	GTCCAGAAGAACTCCATCCTGCTACTTAGCCCGTTTACTTTGGATCTA
**Primers for amplicon generation for next generation sequencing**	
A/H1N1 F	CGCACTCAGAATGAAGTGGA
A/H1N1 R	GCCGAAGGTACCATGTTTCA
A/H3N2 F	CATAGTAGTGCAGAAATGGTTCCGGAGAGA
A/H3N2 R	CATAGTAGTGTTCGGCGTATCTTGACTTGA
A/H5N1 F	CATAGTAGTGTGGAGCAAGACAAATGATGC
A/H5N1 R	CATAGTAGTGCTCCCACTTCATTTGGGAAA
**Primers for real-time RT-qPCR**	
Forward	AAGACCAATCCTGTCACCTCTGA
Reverse	CAAAGCGTCTACGCTGCAGTCC
probe	6-FAM TTTGTGTTCACGCTCACCGTGCC-TAMRA

1 Proctor, D. F. in *Handbook of Physiology* Vol. 1 Ch. 8, 309 (American Physiological Society, 1965).
